# Report of a Case of Cerebral Abscess

**Published:** 1890-10

**Authors:** C. D. Roy

**Affiliations:** House Surgeon Charity Hospital, New York


### Clinical Department.

### REPORT OF A CASE OF CEREBRAL ABSCESS.

BY C. D. ROY, A. B., M. D., HOUSE SURGEON CHARITY HOSPITAL, NEW YORK.

The case in question was so unique in character, both in regard to symptoms, diagnosis and final result, that a report of the same will be of much interest to the readers of THE RECORD.

Thomas M., married, æt 42, was admitted into the Hospital May 21. Family history gave negative results. Previous history—patient had been a hard drinker up to the present accident. Present history shows him to have been perfectly well up to seven weeks ago, when he was hit upon the head with a hammer; did not render him unconscious nor cause him to fall; walked ten blocks and had the wound dressed, which healed in two weeks; has not been able to work since, on account of pain in his head. This extends over the whole head, but is most severe at back and base of brain, of a sharp, shooting character. This became continuously worse, and eight days later he began to get stupid. On the 1st of May he was sent to Bellevue Hospital, where he became delirious; soon recovered sufficiently to return home; come into the hospital on a stretcher in a dull, stupid condition, but conscious when aroused.

Phys. Ex.—Partial paralysis of left side, face, arm and leg. Sensibility diminished in left arm. Scar of wound noted on forehead in median line. Incontinence of urine, bowels normal. Deglutition, speech, pupils normal. Heart, apex beat displaced slightly to left, murmur synchronous with first sound, heard with greatest intensity at apex and transmitted slightly to the left. Lungs apparently normal. Urine alkaline. Temperature 98, pulse 120, respirations 22.

On May 25 a consultation was held and the following conditions found to exist: Complete left hemiplegia, face, arm, leg and tongue. Arm most affected. Planter reflexes exaggerated on left side. Diminished cutaneous sensibility over left arm, face and trunk. Pupils equal. No ocular paralysis except when eyes are at rest, when right eye divulges. Dr. Peterson, the neurologist, diagnosed abscess of brain, which caused pressure on the motor area of right side. They all concurred and decided to operate the next day. The operation was performed by Dr. Kelly, assisted by Dr. Peterson. The scalp and face were shaved and antiseptic preparations made. An incision was made about three inches in length from the median line across the fissure of Rolando towards the ear. The skull having been laid bare, the point was located over the motor arlea, and the bone entered with one and a half inch trephine. When the button of bone was removed, there was great bulging of the grey substance. The opening was enlarged with the bone forceps going anteriorly. The dura mater was opened with scissors and brain substance exposed. Wier's spray apparatus played on wound with a two and a half per cent solution of carbolic acid during the whole operation. Exploratory trocar and carula were introduced to the depth of one inch and nothing evacuated except broken down brain substance. Opening again enlarged anteriorly and another puncture made into the ascending parietal convolution, with same result. After consultation the trocar was again introduced downward and forward to the depth of two inches, and upon aspirating six ounces of pus was removed. The necrossed brain tissue was removed with a Volkman spoon and a drainage tube introduced into the abscess cavity. The scalp wound was then closed and an antiseptic dressing applied. The patient's pulse was very poor throughout the whole operation, and he died six hours after the operation. Had the condition been recognized sooner, the result would have perhaps been more satisfactory, but as it was, an operation was his only hope.

